# Hydrocarbon Removal by Two Differently Developed Microbial Inoculants and Comparing Their Actions with Biostimulation Treatment

**DOI:** 10.3390/molecules25030661

**Published:** 2020-02-04

**Authors:** Joanna Brzeszcz, Piotr Kapusta, Teresa Steliga, Anna Turkiewicz

**Affiliations:** 1 Department of Microbiology, Oil and Gas Institute–National Research Institute, ul. Lubicz 25A, 31-503 Krakow, Poland; anna.turkiewicz@inig.pl; 2 Department of Reservoir Fluid Production Technology, Oil and Gas Institute–National Research Institute, ul. Lubicz 25 A, 31-503 Krakow, Poland; teresa.steliga@inig.pl

**Keywords:** defined mixed culture, undefined community, biostimulation/bioaugmentation, total aliphatic hydrocarbons (TAHs), polycyclic aromatic hydrocarbons (PAHs), toxicity tests

## Abstract

Bioremediation of soils polluted with petroleum compounds is a widely accepted environmental technology. We compared the effects of biostimulation and bioaugmentation of soil historically contaminated with aliphatic and polycyclic aromatic hydrocarbons. The studied bioaugmentation treatments comprised of the introduction of differently developed microbial inoculants, namely: an isolated hydrocarbon-degrading community C1 (undefined—consisting of randomly chosen degraders) and a mixed culture C2 (consisting of seven strains with well-characterized enhanced hydrocarbon-degrading capabilities). Sixty days of remedial treatments resulted in a substantial decrease in total aliphatic hydrocarbon content; however, the action of both inoculants gave a significantly better effect than nutrient amendments (a 69.7% decrease for C1 and 86.8% for C2 vs. 34.9% for biostimulation). The bioaugmentation resulted also in PAH removal, and, again, C2 degraded contaminants more efficiently than C1 (reductions of 85.2% and 64.5%, respectively), while biostimulation itself gave no significant results. Various bioassays applying different organisms (the bacterium *Vibrio fischeri*, the plants *Sorghum saccharatum*, *Lepidium sativum*, and *Sinapis alba*, and the ostracod *Heterocypris incongruens*) and Ames test were used to assess, respectively, potential toxicity and mutagenicity risk after bioremediation. Each treatment improved soil quality, however only bioaugmentation with the C2 treatment decreased both toxicity and mutagenicity most efficiently. Illumina high-throughput sequencing revealed the lack of (C1) or limited (C2) ability of the introduced degraders to sustain competition from indigenous microbiota after a 60-day bioremediation process. Thus, bioaugmentation with the bacterial mixed culture C2, made up of identified, hydrocarbon-degrading strains, is clearly a better option for bioremediation purposes when compared to other treatments.

## 1. Introduction

Hydrocarbon pollution is still one of the most important issues affecting both natural and human-transformed environments [1,2,3]. The last huge oil spill on Brazilian beaches is another example of this significant problem [4]. Consequently, several techniques are employed for the restoration of impacted ecosystems. Bioremediation is a method which is chosen very often, because it is relatively inexpensive and is generally believed to have minimal unintended side effects [5]. Bioremediation of soils contaminated with petroleum hydrocarbons is usually based upon two approaches: biostimulation (an addition of the appropriate nutrients and/or electron acceptors to stimulate the degradation capacity of the indigenous soil microorganisms) and bioaugmentation (an inoculation of soil with high numbers of autochthonous or allochthonous hydrocarbon-degrading microorganisms) [6]. The results obtained in different studies advocate the application of either the former [7,8,9] or the latter method [10,11,12]. Numerous microorganisms possess an ability to use hydrocarbons as the sole source of carbon and energy, but only representatives of a few genera, notably *Mycolicibacterium* (formerly included in *Mycobacterium* genus), *Pseudomonas*, and *Rhodococcus*, are capable of degrading both aliphatic and aromatic hydrocarbons [13]. Since crude oil is a complex mixture of various hydrocarbons and their derivatives, its biodegradation clearly requires the action of different microorganisms [14]. Nutrient amendments may lead to an unspecific stimulation of various soil microorganisms [15], not only hydrocarbon degraders, which in turn can possibly slow down remediation processes. On the other hand, bioaugmentation based on isolating autochthonous (indigenous) hydrocarbon-degrading strains, and then growing them to high densities and finally inoculating the same soil environment they were derived from, yielded very good results [16,17,18]. This approach, though effective, is time- and labor-consuming. Thus, bioaugmentation is performed much more frequently with an application of allochthonous microorganisms [19,20,21,22,23,24,25]. However, in this case, additional factors make the whole process much more complicated. First, not all hydrocarbon degraders are equally suitable to be included in degradative inoculants. For example, some are considered opportunistic pathogens (like *Burkholderia cepacia* [26] or *Pseudomonas aeruginosa* [27,28]) and belong to biosafety level classification 2. Therefore, their field-scale use would pose a serious risk to environmental and human health [29]. The second issue to be addressed is the proper selection of strains. Bacosa et al. [30] showed that some bacterial members of the hydrocarbon-degrading consortium were initially inhibited by the presence of aromatic hydrocarbons and seemed not to be active in hydrocarbon degradation but utilized the metabolic products. In oxygen-limiting conditions, bioaugmentation with the strain *Rhodococcus erythropolis* T902.1 gave better results than the biostimulation treatment [31], while under harsh environmental conditions (high hydrocarbon load and low moisture content), the action of *Mycolicibacterium frederiksbergense* IN53 (a K-strategist) was superior to that of *Acinetobacter* sp. IN47 (an r-strategist) [32]. Finally, one should consider what the fate will be for the introduced non-indigenous microbes and how they will affect indigenous microbiota. This also seems to be dependent on the selected organism. Some studies suggest that augmented strains persist in their new environments [23,33,34], whereas others report the inability of non-native bacteria to compete with indigenous microbiota for a longer period of time [12,31].

In this study, we prepared two hydrocarbon-degrading microbial inoculants (an undefined community C1 and a defined mixed culture C2), tested how they perform in soil polluted with petroleum hydrocarbons with unusually high polycyclic aromatic hydrocarbon (PAH) content, and compared their influence with the action of indigenous microbiota. Apart from examining their biodegradative efficiency by chromatographic analyses, we also used a set of toxicity tests (biotests) to confirm that the remediation process did not leave toxic intermediates. We also checked whether the addition of various allochthonous microorganisms present in the C1 and C2 can change the native microbial community.

## 2. Results

### 2.1. Structure of Hydrocarbon-Degrading Community C1

The genus-level taxonomic structure of bacterial community C1 is dominated by *Alcaligenes* and *Pseudochrobactrum* (Table 1). Other members of the C1 are *Aquamicrobium*, *Enterococcus*, *Brevundimonas*, and *Alkaliphilus*. Amplicon sequence variants (ASVs) assigned as *Clostridium sensu stricto* 16, *Clostridioides*, *Melissococcus*, and *Leucobacter* were found in the C1. However, their relative abundances were relatively low (1–2%).

### 2.2. Hydrocarbon Removal 

**Figure 1:**
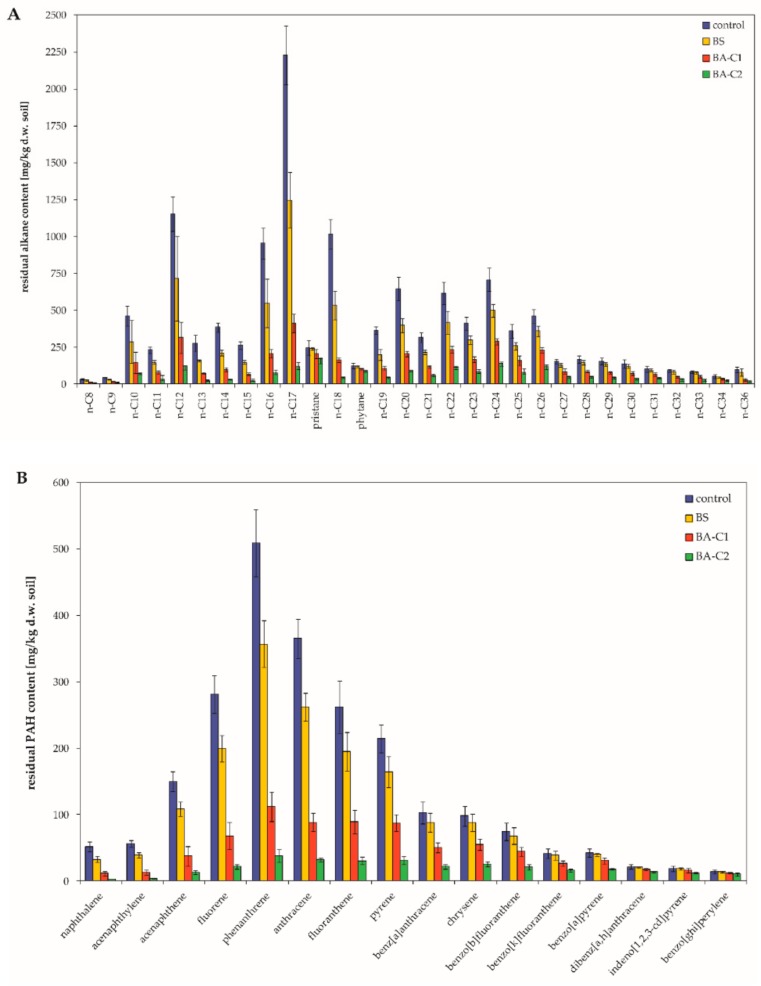
Residual content of (**A**) alkanes and (**B**) polycyclic aromatic hydrocarbons (PAHs) in the analyzed microcosms. Control: untreated microcosms, BS: biostimulated microcosms, BA-C1 and BA-C2: microcosms bioaugmented with the bacterial community C1 and the mixed culture C2, respectively. Data are the mean values ± SD (*n* = 4).

Abiotic hydrocarbon removal (HgCl_2_-treated soil sample) was negligible (approximately 1%); presumably, it was a result of chemical degradation during the incubation period. Untreated soil samples (natural attenuation) also exhibited a marginal decrease (less than 1%) of total petroleum hydrocarbons (TPH) in comparison to the abiotic control. Therefore, these microcosms (control) were regarded as references for other treatments. After a 60-day incubation, the total aliphatic hydrocarbon (hereinafter referred to as TAH) level was 17 467.2 ± 1049.5 mg/kg dry weight of soil (further d.w. soil) in the control microcosms (Table 2), whereas the value of this parameter in BS (biostimulation), BA-C1 (bioaugmentation with the hydrocarbon-degrading community C1), and BA-C2 (bioaugmentation with the mixed culture C2, containing only well-characterized hydrocarbon degraders) treatments was lowered, respectively, to 11 372.0 ± 699.8, 5 290.9 ± 297.7 and 2310.3 ± 126.2 mg/kg d.w. soil (Table 2). In the case of the BA-C1 and BA-C2 treatments, this value corresponded to a significant decrease of 69.7% (*p* < 0.01) and 86.8% (*p* < 0.0001), respectively. Moreover, inoculation with the C2 resulted in significantly higher TAH removal compared to the BS treatment (reduction of 34.9%; *p* < 0.0005) and bioaugmentation with the C1 (*p* < 0.05). The residual total polycyclic aromatic hydrocarbon (PAH) level was 2785.6 ± 162.4 mg/kg d.w. soil in the control, which was reduced to 2120.8 ± 118.6 (a reduction of 23.9%), 988.7 ± 54.3 (a reduction of 64.5%), and 411.6 ± 21.9 mg/kg d.w. soil (a reduction of 85.2%) in the BS, BA-C1, and BA-C2 treatments, respectively (Table 2). However, only bioaugmentation with the mixed culture (BA-C2) significantly promoted PAH degradation compared to the control (*p* < 0.001) and the BS (*p* < 0.005). Contents of individual *n*-alkanes, as well as PAHs, decreased in all treated microcosms compared to the untreated ones (Figure 1). In every microcosm, short- and medium-length saturated aliphatic compounds (*n*C_8_–*n*C_22_) were more efficiently degraded than higher homologues (Table 2), and this was also true for low molecular weight PAHs (Table 2). Compared to the control, both ∑*n*C_8_-*n*C_22_ and ∑ two and three PAH contents significantly decreased by 74.9% and 76.7% in BA-C1 (*p* < 0.01, *p* < 0.05) and by 90.4% and 92.1% in BA-C2 (*p* < 0.0001, *p* < 0.005) under these experimental conditions. Furthermore, when ∑*n*C_8_-*n*C_22_ changed, there were significant differences between the BA-C2 and other treated microcosms (BS and BA-C1, *p* < 0.001, *p* < 0.05). N and P addition (BS) also enhanced the degradation of higher *n*-alkanes since the ∑*n*C_23_-*n*C_35_ content was lowered by 21.7%. However, greater reductions were noted for the bioaugmented soils (BA-C1: 52.4% and BA-C2: 74.6%). The introduction of allochthonous bacteria strongly affected four- and five-ring PAH degradation and led to a decrease of 53.1% and 79.3% in the BA-C1 and BA-C2 microcosms, respectively. Stimulation of native PAH degraders yielded only 18.0% degradation of those aromatic compounds. In turn, application of the C2 had a significant effect on the removal of both *n*C_23_-*n*C_35_ and four- and five-ring PAHs compared to the control (*p* < 0.005, *p* < 0.05) and the BS treatment (*p* < 0.01, *p* < 0.05). Higher molecular weight PAHs were more resistant to degradation. The range of six-ring PAH depletion was 3.35%–31.4%, and the highest efficiency was found for the BA-C2 treatment. However, the observed reductions were not statistically significant in any treatments (Table 2). The treated samples also demonstrated reduced values of *n*C_17_/pristane and *n*C_18_/phytane ratios compared to the control (Table 3). The lowest values of these parameters were noted for BA-C2 (Table 3). Along with the biodegradation progress, the mentioned ratios should be lower. The more decreased the values of the *n*C_17_/pristane and *n*C_18_/phytane ratios, the more efficient the bioremediation.

As mentioned above, the efficacy of hydrocarbon removal differed between treatments, and the greatest degradation yields of both *n*-alkanes and PAHs were noted for the BA-C2 treatment. These observations suggest higher microbial activity towards hydrocarbons in the BA-C2 microcosms than in the other ones. Thus, biostimulation combined with the introduction of the mixed culture comprising of tailored hydrocarbon-degrading strains (C2) was the most effective bioremediation strategy under these experimental conditions. On the other hand, biostimulation itself was much less effective, and the observed decrease in PAH content was, surprisingly, not statistically significant.

### 2.3. Toxicity and Mutagenicity Assessment 

**Figure 2:**
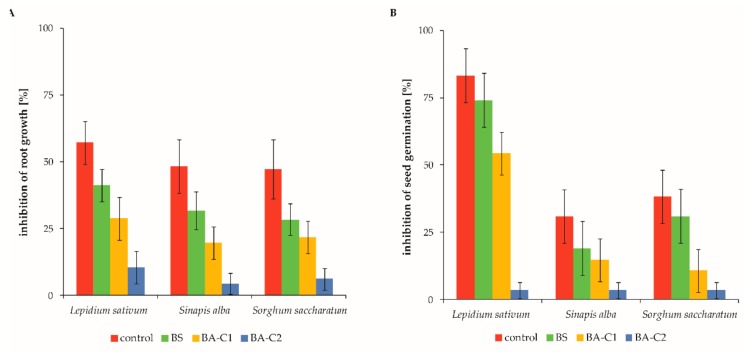
Inhibition of (**A**) root growth and (**B**) seed germination in the analyzed soil microcosms. The data are presented as the mean values ± SD (*n* = 4). Control: untreated microcosms, BS: biostimulated microcosms, BA-C1 and BA-C2: microcosms bioaugmented with the bacterial community C1 and the mixed culture C2, respectively.

After a 60-day bioremediation process, the effectiveness of the toxicity and mutagenicity removal was assessed. The initial (at the beginning of process) toxicity and mutagenicity of the unmodified soil was similar to that found for the control soils at the end of the bioremediation process. Also, in this case, the control microcosms were regarded as references for the BS, BA-C1, and BA-C2 treatments. Based on the obtained results, it is clear that the soil of the control microcosms was toxic to plants (Figure 2), animals, and microorganisms (Table 4).

For the control soils, the noted inhibition of seed germination ranged between 30.9% and 83.2%, depending on the tested organism (Figure 2A), whereas more than 45% of root elongation was inhibited for each analyzed plant (Figure 2B). The results of the Phytotoxkit tests showed that each treated soil revealed lower levels of inhibition of both seed germination and root length growth than the control microcosms (Figure 2). The range of phytotoxicity responses varied for different plant species. However, the trend—the lower content of residual pollution, the lower the inhibition of both germination and root elongation—was observable for *Sorghum saccharatum*, *Lepidium sativum*, and *Sinapis alba* (Figure 2). Among these species, *Lepidium sativum* was the most sensitive to residual contamination after a 60-day bioremediation processes (Figure 2). The results obtained for the mentioned organisms are discussed here in detail, but these observations were also true for other plants. Biostimulation decreased the inhibition of root length growth and seed germination by 16.0% and 7.2%, respectively (Figure 2). Both used bioaugmentation variants also improved the soil quality by reducing phytotoxicity in comparison to the control microcosms. Inhibition of root growth and seed germination was lowered by 28.5% and 28.9% after introduction of the community C1 (BA-C1, Figure 2). However, drastic decreases of both parameters were detected for the BA-C2 soils since inhibition levels of root elongation and germination declined, respectively, by 46.7% and 79.8% (Figure 2). As mentioned above, each treatment varied in its effectiveness in phytotoxicity reduction. Generally, the less contaminated the soil, the less it was phytotoxic.

Direct contact with the control soils led to a 54.6% ostracod mortality (Table 4). However, there was improvement of ostracod survival and its growth in the soils after treatments (BS, BA-C1, BA-C2). The range of neonate mortality was 17.0%–40.3%, whereas growth inhibition was 20.4%–44.5% (Table 4). Compared to the control soils, there was a decrease in acute toxicity by 14.3%, 21.5%, and 37.6% in the BS, BA-C1, and BA-C2 microcosms, respectively. Growth inhibition also differed between the samples, and the lowest value was found for the BA-C2 soils (Table 4). As reported in Section 2.2., the residual hydrocarbon content differed between the soils. These findings show that the less contaminated the soil, the less toxic it is to animal biota. However, some toxic effects were still noticed also in the microcosms characterized by the lowest values of ostracod survival and growth inhibition (BA-C2). Exposure to the contaminants of BA-C2 soils caused approximately 20% of applied animals to not survive and grow properly (Table 4). 

The Microtox Solid Phase Test-based toxicity, expressed as toxicity units (TU), was high in the control soils (Table 4). Despite their differences, each treatment resulted in a reduction of the toxicity to microorganisms (Table 4). Compared to the control soil, TU decreased by 20.3%, 52.2%, and 95.8% in the BS, BA-C1, and BA-C2 treatments, respectively (Table 4). Thus, a less toxic effect was achieved in the soil inoculated with the mixed culture C2. These results also confirm that toxicity reduction is correlated with the effectiveness of contaminant removal.

The mutagenicity ratio is expressed as a ratio of the number of induced revertants to the number of spontaneous ones. The value of this parameter for each soil is collected in Table 4. According to the procedure of the applied test, the sample was considered mutagenic when its mutagenicity ratio was ≥ 2. The obtained results show that potentially mutagenic and carcinogenic compounds were present in the control, BS, and BA-C1 soil extracts but not in the those from the BA-C2 microcosms (Table 4). The control soils exhibited the highest level of mutagenicity, whereas each treated soil demonstrated lower values of the above-mentioned ratio (Table 4). Compared to the control, there was a 26.05%, 49.3%, and 91.5% decrease in the BS, BA-C1, and BA-C2 microcosms, respectively. Thus, the greatest reduction of this parameter was noted in BA-C2 soils, and the thread associated with the presence of analyzed mutagens in this soil was relatively low. Therefore, the approach based on bioaugmentation with the mixed culture that consisted of defined degraders, which caused mutagenicity removal, was the most effective under these experimental conditions.

The above-mentioned observations indicate that each hydrocarbon bioremediation strategy improved soil quality by reducing toxicity, as well as genotoxicity. Among all studied approaches, only the variant including the introduction of the C2 was the most potent treatment.

### 2.4. Soil Community Structure after Treatments

The effects of various bioremediation strategies on the bacterial community structure were evaluated by using 16S rDNA sequencing. The differences in microbial diversity between the treatments were confirmed by several indices, which were computed for the amplicon sequence variants (ASVs; Table 5). The observed richness, ACE, and Chao-1 indices were higher in the control than in all treated soils (Table 5). The lowest values of these parameters were found for the BA-C1 soil (Table 5). This observation indicates that both biostimulation and bioaugmentation approaches decreased bacterial richness. After a 60-day bioremediation process, microbial diversity with the BA-C2 treatment increased compared to the control soil, whereas other treatments led to a decrease in values for both the Shannon and Simpson indices (Table 5). The greatest reductions of both parameters were found for the soil bioaugmented with the community C1 (BA-C1; Table 5). 

**Figure 3:**
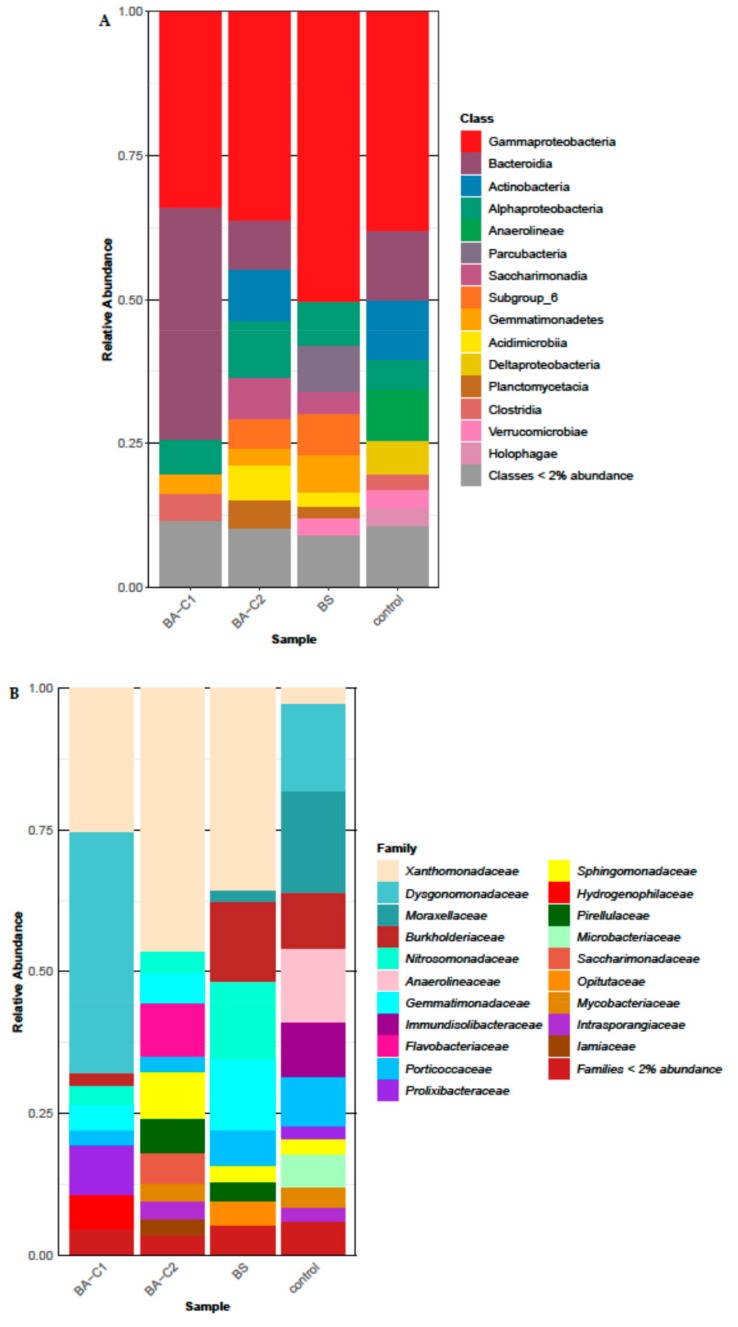

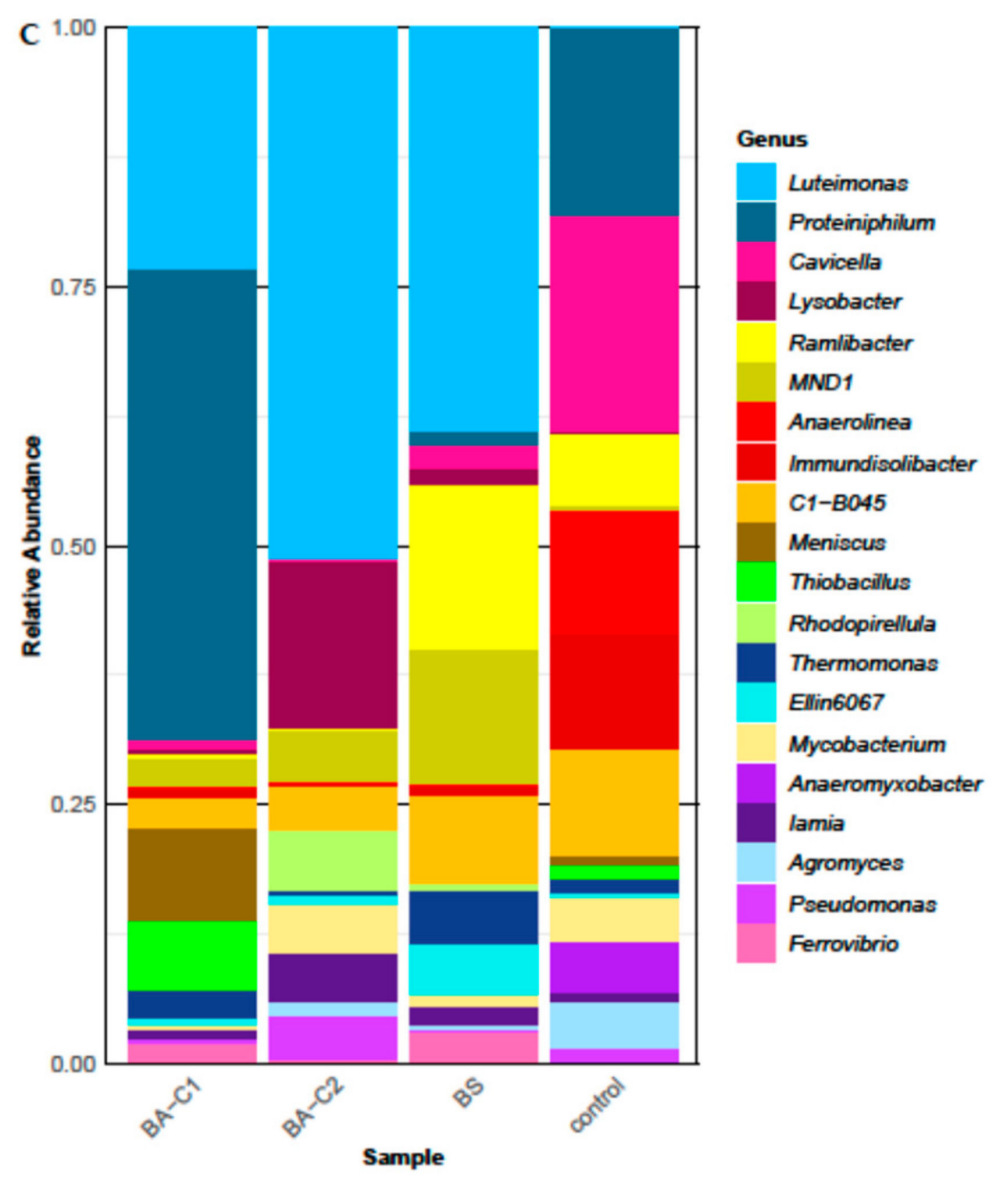
Soil bacterial community structure at the (**A**) class and (**B**) family level after 60-day bioremediation treatments. (**C**) Twenty of the most abundant genera in the analyzed communities. Please note that the sum of the relative abundances (100%) shown here refers only to the most abundant genera for better visualization of the results. The real values are presented in Section 2.4. Control: untreated microcosms, BS: biostimulated microcosms, BA-C1 and BA-C2: microcosms bioaugmented with the community C1 and the mixed culture C2, respectively.

The community structure changed between the treatments (BS, BA-C1, BA-C2) and the control after 60-day bioremediation at the analyzed taxonomic levels (Figure 3). We have skipped analyzing the abundance of phyla and classes because significant differences were seen chiefly in the lower taxa. However, three of the most dominant classes in the control samples—Anaerolineae (9.1%), Deltaproteobacteria (5.8%), and Holophagae (3.2%)—were almost absent in all three treated soils (Figure 3A). This observation is in a good accordance with the substantial decrease of petroleum hydrocarbons in the biostimulated and bioaugmented samples, which, in turn, likely changed the soil environment from mostly anoxic to aerated.

The structure of the soil bacterial community differed between the samples at the family level (Figure 3B). The differences in relative abundance among the treated microcosms were analyzed in comparison to the control. The taxa *Anaerolineaceae*, *Immundisolibacteraceae*, *Moraxellaceae, Microbacteriaceae*, and *Porticoccaceae* were detected in much higher proportions in the untreated sample (respectively, 12.9%, 9.7%, 17.8%, 5.9%, and 8.7%,) than in the BS (respectively, 0.2%, 0.7%, 2.0%, 0.6%, and 6.3%), BA-C1 (respectively, 0.1%, 0.9%, 0.2%, and 2.8%), and BA-C2 (respectively, 0.05%, 0.1%, 0.1%, 1.5%, and 2.8%) samples (Figure 3B). The decrease of these families can be at least partially attributed to the removal of petroleum hydrocarbons in all three treatments. In turn, each treatment enhanced different bacterial families (Figure 3B). Amplicon sequencing revealed an increase of *Burkholderiaceae* (14.1%), *Nitrosomonadaceae* (13.7%), *Gemmatimonadaceae* (12.6%), and *Opitutaceae* (4.7%) in the BS in comparison to the control (respectively 10%, 1.3%, 0.4%, and 2%) (Figure 3B). Except for the *Opitutaceae* taxon, the range of relative abundance of the other families was 1.4%–5.5% in the bioaugmented soil samples. A marked increase in the proportion of *Dysgonomonadaceae* (42.5%), *Prolixibacteraceae* (8.6%), and *Hydrogenophilaceae* (6.1%) occurred in the BA-C1 community compared to the control (15.4%, 2.2%, and 1.2%, respectively). Greater relative abundance of the *Flavobacteriaceae* (9.3%), *Sphingomonadaceae* (8.3%), *Pirellulaceae* (6.0%), *Saccharimonadaceae* (5.3%), and *Iamiaceae* (3.0%) families was found in the BA-C2 treatment than in the control (not detected, 0.5%, 0.3%, 0.02%, and 0.7%, respectively). All treatments resulted in an increased abundance of *Xanthomonadaceae* (Figure 3B), since its relative abundance was 46.3%, 35.5%, 25.2%, and 2.7% in the BA-C2, BS, BA-C1, and control treatments, respectively. Statistically significant changes were recorded at the genus level (*p* < 0.0001). 

All three bioremediation approaches, however, caused a different composition of microbial genera when referring to those of the highest abundance (the 20 most abundant, Figure 3C). Among them, the *Luteimonas* taxon was one of the most dominant in all treated soils (Figure 3C), and its relative abundance in the whole analyzed community structure of the BS, BA-C1, and BA-C2 microcosms was respectively 14.9%, 16.4%, and 18.7%. In turn, its abundance in the control microcosm was 0.01%. Apart from this genus, the bacterial community of the BS soil was dominated by *Ramlibacter* (6.1%), *MND1* (4.9%), *Thermomonas* (1.9%), and *Ellin6067* (1.9%). In turn, the most dominant taxa in the BA-C2 soil included *Lysobacter* (5.8%), *Salinimicrobium* (2.1%), *Rhodopirellula* (2.1%), *MND1* (1.8%) and *Mycobacterium/Mycolicibacterium* (1.8%), while the *Proteiniphilum* (32.2%), *Luteimonas* (16.4%), *Meniscus* (6.4%), *C1-B045* (2.1%), and *MND1* (1.9%) taxa were the predominant taxa in the other bioaugmented soil (BA-C1). The major genera (with an abundance greater than 5%) in the bacterial community of the control soils were: *Cavicella* (11.3%), *Proteiniphilum* (9.9%), *Anaerolinea* (6.5%), *Immundisolibacter* (6.1%), and *C1-B045* (5.6%). Obligate anaerobes, like *Anaerolinea* (6.5%), *Anaeromyxobacter* (2.6%), *Ruminiclostridium* (1.1%), *Longilinea* (0.65%), and *Geobacter* (0.63%) were found in the control microcosm; however, they significantly diminished (> 0.2% *Ruminiclostridium* in the BA-C2 soil; >0.15% *Anaerolinea* and *Geobacter* microorganisms in all amended soils), or even perished (*Anaeromyxobacter*, *Ruminiclostridium,* and *Longilinea*) in all treated microcosms. Furthermore, some aerobic or facultative anaerobic taxa present in the non-amended soil, such as *Immundisolibacter* (6.1%), *Altererythrobacter* (1.1%), and *Pseudoxanthomons* (1.0%), were clearly outcompeted (relative abundance less than 0.5% in the treated microcosms) by other microorganisms. On the contrary, some genera greatly increased their abundance. For example, inoculation with the community C1 increased *Proteiniphilum* and *Thiobacillus* since their relative abundance changed, respectively, from 9.9% to 32.2% and 0.8% to 4.7% in the control and the BA-C1.

Among the genera introduced to the soil as the mixed culture C2 (the BA-C2 sample), only the *Mycobacterium/Mycolicibacterium* and *Pseudomonas* (1.55%) taxa were represented in the community structure among 20 the most abundant genera (Figure 3C). The abundance of the other taxa introduced as the C2—*Gordonia*, *Dietzia*, *Rhodococcus,* and *Arthrobacter—*was less than 1% (jointly) in the BA-C2 soil. On the other hand, the mentioned genera were more abundant in the BA-C2 microcosms than in the other microcosms (data not shown). Besides the *Gordonia* and *Rhodococcus* genera, the other taxa were also present in the untreated samples. This result suggests that representatives of these genera may have not only participated in hydrocarbon degradation but also sustained competition from other bacteria for the time required to degrade the pollutants. This was not true in case of hydrocarbon degraders present in the community C1, since none of them seemed to persist 60 days after they had been introduced to the contaminated soil.

### 2.5. Gene Expression of Catabolic Genes

To determine if bacteria introduced as the mixed culture C2 participated in hydrocarbon degradation, we investigated the taxonomic distribution of some functional genes expressed in the BA-C2 microcosms. Transcripts coding for alkane monooxygenase (AlkB) were assigned to several genera belonging to the Gammaproteobacteria and Actinobacteria classes (Figure S1). A great proportion of these transcripts was mapped to *Pseudomonas* (Supplementary Materials, Figure S1). Interestingly, some of these transcripts were similar to those from the *Mycobacteriaceae* family (*Mycobacterium/Mycolicibacterium)* and the *Gordonia* and *Arthrobacter* genera (Supplementary Materials, Figure S1).

## 3. Discussion

In this study, the effects of biostimulation and biostimulation combined with the bioaugmentation of soil contaminated with aliphatic hydrocarbons and a high load of PAHs were compared. Bioaugmentation is regarded as a strategy to enhance bioremediation via the introduction of a microbial agent pre-adapted to the environmental conditions of the contaminated site. There are two main criteria that should be considered for the selection of a microbial formula—the hydrocarbon-tailored catabolic potential of microorganisms, their functional activity, and their persistency under the given conditions. Therefore, the choice of microorganisms for inoculation should not be indiscriminate. This is quite easy to achieve when indigenous microorganisms are chosen for bioremediation purposes [16,17,18,35]. However, it should be stressed that under the term “bioaugmentation”, one should consider only bioaugmentation combined with parallel nutrient amendments [36], as we did here and in our earlier studies [16,17]. We applied two different approaches to obtain allochthonous inoculants, namely a hydrocarbon-degrading community as well as a mixed culture. They both were constructed with bacteria originating from long-term contaminated soils with crude oil or diesel fuel, which are composed of aliphatic and aromatic hydrocarbons. The mixed culture C2 was developed via the careful selection of bacterial strains with identified metabolic capabilities towards both *n*-alkanes and monocyclic and polycyclic aromatic compounds. This approach (that is, constructing a defined mixed culture with well-characterized metabolic activities) is a widely accepted solution [19,20,37,38]. In turn, the community C1 was obtained via enrichment from soils with a long history of contamination with crude oil and a random selection of hydrocarbon degraders. As determined by Miseq sequencing, the C1 contained a myriad of different bacteria but was dominated by few genera. Among these taxa, *Alcaligenes*, *Pseudochrobactrum, Aquamicrobium*, *Enterococcus*, *Brevundimonas*, and *Alkaliphilus* presented the highest abundance. Representatives of the genera *Alcaligenes* [39,40], *Aquamicrobium* [41], *Brevundimonas* [42,43], and *Enterococcus* [44,45] were reported to have hydrocarbon-degrading abilities, while others were not. Thus, this is evidence that both the enrichment method and randomly picking colonies grown on crude-oil-coated agar plates, apart from yielding hydrocarbon degraders, may produce some unwanted “companions” (e.g., *Clostridium sensu stricto* 16 and *Clostridioides*). In this case, cooperation in the metabolic degradation of pollution should have naturally selected the microbial population, as shown recently [46,47]. On the other hand, some of these members might have been considered not necessarily beneficial since some representatives of the *Brevundimonas* [48] and *Enterococcus* [49] genera are also known as opportunistic pathogens. The composition of the community C1 was analyzed but the detailed catabolic activities of the C1 and its individual members were not examined. However, the observed removal of alkanes and PAHs in bioaugmented microcosms (BA-C1, BA-C2) indicated that both microbial inoculants were composed of organisms capable of degrading various structurally diversified hydrocarbons, even though a considerable increase in the relative abundance of the indigenous potential PAH-degrader *Proteiniphilum* [50] was noted in the former microcosms (BA-C1). Furthermore, in our study, the numbers of introduced allochthonous microbes were relatively low (not exceeding 10^6^ colony forming units/g d.w. soil (cfu/g d.w. soil)) and were only slightly higher, if at all, than the numbers of indigenous microbiota (8.7 ± 4.3 × 10^5^ cfu/g d.w. soil). These observations fully support the idea of bioaugmentation as a bioremediation strategy and are contrary to some other reports [22,25].

The presented chromatographic results clearly indicate that the introduction of the mixed culture was a more efficient bioremediation approach than the other studied bioaugmentation variant, as well as biostimulation sensu stricto. Additionally, only inoculation with the C2 treatment resulted in almost complete removal of nearly the total range of the analyzed compounds. In turn, long-chain *n*-alkanes, as well as four- and five-ring PAHs, were poorly degraded in the biostimulated soil and less efficiently in the one bioaugmented with the community C1. Furthermore, the BA-C2 treatment seemed to be a suitable solution for the restoration of hydrocarbon-contaminated soil with a high load of PAHs since it led to the most effective elimination of both toxic and genotoxic effects. The members of the mixed culture were selected according to their hydrocarbon-degrading capabilities. Therefore, the mentioned bacteria might have predominantly participated in the conversion of toxicants to less or non-toxic intermediates or by-products. In turn, the biodegradation efficiency of the other bioaugmentation option was limited. Thus, the presence of residual pollutants might have posed a threat to living organisms, as revealed by the bioassay results. It was shown that a decrease of hydrocarbon content in bioremediation treatments does not always infer a reduction in toxicity [51,52]. Mineralization of hydrocarbons in aerobic conditions results in formation of non-toxic substances, such as CO_2_ and water. Some transformations that do not lead to complete metabolism of the parent compound are inevitable in a complex system such as polluted soil [52,53]. In the case of PAHs, incomplete metabolism (especially in the oxidation step) of an aromatic structure may lead to formation of more polar and mobile intermediates, which may be as toxic as a parent compound or even more so [54]. The relaxed substrate specificity of aromatic ring-hydroxylating dioxygenases is the reason for oxygenated PAHs (oxy-PAHs) generation. These compounds are regarded as intermediates but also “dead-end” metabolites [55]. Moreover, identification of the novel product of pyrene degradation in post-remediated soil [53] indicates that our current knowledge on hydrocarbon biotransformation is still limited since it is mainly based on the information gathered from studies which were performed using a single, model compound. In turn, degradation of a complex mixture, such as a PAH mixture, may also involve antagonistic effects associated with the use of an unusual pathway that is not normally used by the organism in the degradation of a single compound [56]. Thus, bioremediation of soil contaminated with hydrocarbons may lead to generation of unknown compounds with unknown toxicity. It was demonstrated that products of PAH transformation were accumulated in several soil remediation studies [52,54,57] and they may be responsible, at least in part, for an increase in toxicity observed during remedial treatments of contaminated soils ([52,54] and references cited therein) since these compounds are not degraded concomitantly with PAHs. Despite recent progress in analysis of compounds generated during PAH biodegradation [53], it is challenging to monitor the presence and concentration of these substances. Therefore, bioassays are an alternative to evaluate hazards before, during, and after remediation. However, it should be highlighted that the toxicity/mutagenicity response is an effect of the total load of soil toxicants and potential interferences among them. The abilities of the microorganisms to (1) mineralize the target substances or (2) generate products to such low levels that they are safe from the perspective of risk, will determine the efficacy of bioremediation. In this study, potential soil toxicity after treatments was assessed by a set of bioassays. In each bioremediation variant, removal of hydrocarbon content was associated with a reduction in toxicity and these observations are consistent with those of Płaza et al. [58].

Our findings also prove that inoculation using pre-selected isolates with the ability to metabolize both *n*-alkanes and PAHs is a better option than the enrichment approach for bioremediation, although solutions like the immobilization of bacterial cells may increase the degradation rate of hydrocarbons [59,60]. Most PAHs, as well as *n*-alkanes, were degraded most efficiently in the BA-C2 microcosms. Additionally, both the toxicity and mutagenicity assessment revealed that BA-C2 microcosms presented the highest levels of soil reclamation among all treatments, although some level of toxicity was detected due to residual PAH content. The mixed culture C2 produced a significant increase in *n*-alkane and PAH biodegradation efficiency and outperformed the expectations placed upon it. The proper selection of bacterial strains is always a crucial step, and well-known hydrocarbon degraders may succeed or fail depending on the environment they are introduced to [11,32]. In favorable conditions, bioaugmentation with even a single strain resulted in the effective removal of hydrocarbons [61] and even within one species, one could find a difference among individual strains in this regard [62].

There are many reports showing the success of bioaugmentation to remediate hydrocarbon-polluted soils [17,23,32,62,63,64]. However, its positive effects may be limited to the early stage of treatments [12,24]. On the other hand, in some cases this strategy is not the best remedial option [65]. In this study, we showed the advantage of bioaugmentation over biostimulation as well as the advantage of the mixed culture C2 (defined) over the community C1 (undefined). The latter observations are consistent with the findings of other authors [66,67]. Microbial strains growing and evolving together, as in the C1, should be more productive as a group since some adapt to utilizing the by-products of other members. However, in our study the total number of hydrocarbon degraders present in the C2 was apparently higher than in the C1, because each strain of the C2 exhibited wide hydrocarbon-degrading capabilities, while presumably only some members of the community C1 could have been regarded as true hydrocarbon degraders. Moreover, the antagonism between bacterial hydrocarbon-transforming populations in the C1 during degradation should not be excluded. Festa et al. [67] proved that negative interactions existed within undefined communities even if a single compound (phenanthrene) was degraded and these interactions influenced the final outcome. The other possible advantage of the mixed-culture C2 was that it included both K- (for example *Mycolicibacterium frederiksbergense* IN53) and r-strategists (for example *Pseudomonas* sp. IN132), that is, fast- and slow-growing organisms in roughly similar numbers (within the same order of magnitude), after growing in a nutrient-rich medium. In the undefined community C1 the K-strategists were virtually outgrown by r-strategists as judged by the low (1%) abundance of slow-growing *Leucobacter* species. Our results are contrary to other studies in which an excellent efficiency of hydrocarbon-degrading communities/consortia [30,68] was clearly presented. However, those communities/consortia were grown exclusively on mineral media supplemented with hydrocarbons and therefore consisted only of hydrocarbon degraders. Sydow et al. [68] demonstrated that members of the hydrocarbon-degrading consortium drastically change their abundance upon exposure to different carbon sources (various hydrocarbons and biodiesel), whereas the overall structural and functional integrity of the consortium was maintained, but was apparently not retained in our undefined community C1. On the other hand, field-scale use usually requires large volumes (hectoliters or even cubic meters) of microbial inoculants and this is usually achieved by growing cells in a nutrient-rich medium or in a mineral medium supplemented with easily accessible water-soluble industrial by-products such as molasses [69]. Furthermore, from this point of view, mixed cultures could be also regarded as effective inoculants for bioremediation purposes [66,70].

We also analyzed how all three treatments affected the indigenous soil microbial community. Since only pooled samples were analyzed, we did not perform a multivariate statistical analysis. Thus, this analysis was primarily used to evaluate the effects of the treatments on the bacterial taxa that were characterized by the highest abundance, as in [12]. We focused mostly on bacterial families and genera because the observed differences were significant on those taxonomical levels. The finding that all treatments strongly affected the anaerobic part of the native community is not unusual, as hydrocarbon removal usually indicates a facilitated mass transfer rate of air (oxygen) to soil (this effect can be also achieved by the prior use of a bulking agent [71]). Moreover, each treatment was performed in aerobic conditions. The native hydrocarbon-degrading population was also impacted since potential hydrocarbon degraders present in the non-amended soil, such as *Immundisolibacter* [72], *Altererythrobacter* [73,74], and *Pseudoxanthomons* [75], were clearly outcompeted by other microorganisms. However, the differences in dominant genera between all treated microcosms (with the exception of the most abundant, *Luteimonas*) and the weak presence of typical hydrocarbon degraders were unexpected. This was also true for the introduced bacteria, among which only *Pseudomonas* and *Mycobacterium*/*Mycolicibacterium* exhibited relative abundances higher than 1.5% in the BA-C2 microcosm, but these genera were also present in the control soil sample, so there is no way to determine whether they were indigenous or non-indigenous. The abundance of the other introduced hydrocarbon degraders was marginal, but the *Gordonia* and *Rhodococcus* genera were present only in soil inoculated with the C2. Moreover, our metatranscriptomic results showed that the *alkB* transcripts assigned to *Mycobacterium*/*Mycolicibacterium*, *Pseudomonas*, *Arthrobacter*, and *Gordonia* were found in the BA-C2 microcosms. These observations suggest that non-native bacteria present in the defined mixed culture sustained competition from indigenous microbiota for at least the time required to remove most of the contaminants; however, they were unable to establish larger populations in their new habitat.

Although we did not study the influence of natural attenuation on hydrocarbon removal in great details, our results show relatively low effectiveness of this approach compared to other reports [65,76,77,78]. In turn, other authors also noticed insufficient effects of naturally occurring processes as a remediation strategy [79,80]. It was suggested that both soil properties and the indigenous soil microbial population determine the biodegradation effect [65]. Natural attenuation occurs in environments, but at least one of following conditions must be fulfilled: (1) it should be the adequate concentration of available P and N, and/or (2) the soil texture should allow bacteria to have facilitated access to hydrocarbons and oxygen. In laboratory conditions, the latter could be achieved by passing the soil through a sieve [76,77,78]. However, this practice was not applied in this study. Other parameters such as soil pH and nutrient availability should also be considered. In this study, the pH of non-amended soil was slightly acidic (6.1) and the C:N:P ratio was not optimal for bacterial requirements (approximately 100:2:0.4). Addition of nutrients and a change in pH value led to improved biodegradation as revealed by hydrocarbon removal in biostimulated soil (BS). This finding also suggests that studied native microorganisms may have the potential to metabolize mentioned compounds, but are not as active in non-optimal conditions as those in the control microcosm. This also confirms the results obtained by Adetutu et al. [79] and Košnář et al. [80]. In our study, the presence of crude oil led to developed anaerobic conditions, in which oxygen transfer was limited. Even performed manipulations (aeration) did not help to change these conditions as indicated by the structure of the bacterial community in the control soil (natural attenuation) after a 60-day incubation. Although the structure of the bacterial community at the beginning (0 day) of the process was not analyzed, the non-amended soil at the end of experiment was rich in obligate anaerobes (e.g., *Anaerolinea, Anaeromyxobacter*, *Ruminiclostridium*, *Longilinea* and *Geobacter*; together more than 11% of all bacteria). The effectiveness of anaerobic hydrocarbon biodegradation is significantly lower than aerobic processes. Our data also proved that the action of the native population of hydrocarbon-degrading microorganisms in the control soil was limited. Light, relatively easily biodegraded *n*-alkanes (*n*C_8_-*n*C_12_, Figure 1A) and low molecular weight PAHs (naphthalene, phenanthrene, Figure 1B) were still present in the 50-year weathered soil pollution. Assuming high effectiveness of indigenous hydrocarbon-degrading populations, only more recalcitrant compounds should be present in the analyzed soil as reported by Gallego et al. [81]. It should be noted that the extent of natural attenuation processes depends also on hydrocarbon bioavailability. The slow desorption from the sorbed to aqueous phase means restricted bioavailability and thereby low extent of hydrocarbon degradation.

## 4. Materials and Methods

### 4.1. Chemicals

Unless otherwise stated all chemicals were of analytical grade and purchased from Avantor Performance Materials (Gliwice, Poland).

### 4.2. Isolation of Hydrocarbon-Degrading Community C1

The hydrocarbon-degrading community (designated C1) used in this study was obtained via enrichment from crude-oil-polluted soils deposited in the weathered waste-pits located in southeast Poland. A total of 10 g of soil was suspended in 90.0 mL of sterile NaCl (0.85%, *w*/*v*) with added sodium pyrophosphate (0.1%, *w*/*v*) and shaken (1 h, room temperature, 150 rpm). Then, 1.0 mL of serial dilutions of the suspension were spread onto the surface of a modified Bushnell-Haas agar plate (1 g/L K_2_HPO_4_, 1 g/L KH_2_PO_4_, 1 g/L NH_4_NO_3_, 0.02 g/L CaCl_2_, 0.05 g/L FeCl_3_, 0.2 g/L MgSO_4_, 20.0 g/L agar, 1 g/L NaCl, final pH 7.0 ± 0.2 supplemented with 1.0 mL SL-10 trace element solution) with sterile crude oil as the sole carbon source. The incubation was performed at room temperature for 20–30 days. After this time, the cultures growing in the presence of hydrocarbons at the highest dilutions were selected as members of the consortium. Additionally, 1.0 mL of serial dilutions of the soil suspension was introduced into 50 mL of the modified Bushnell-Haas broth supplemented with 1% (*v*/*v*) crude oil and kept at 25 °C for 50 days. After hydrocarbon-degrading bacteria had developed under the oil layer, a 1.0 mL aliquot was taken for the consortium. The degradation capabilities of the C1 were confirmed several times by observed growth in mineral medium supplemented with crude oil as the sole source of carbon and energy. The C1 was grown in a BD Difco™ nutrient broth (BD-Poland) supplemented with 0.2% (*w*/*v*) sodium acetate and 1% (*v*/*v*) crude oil to reach a density of 10^8^–10^9^ cfu/mL. The bacterial structure of community C1 was analyzed in this study.

### 4.3. Construction of Mixed Culture C2

Seven non-pathogenic bacterial strains used in this study (Rhodococcus erythropolis IN119, Rhodococcus sp. IN136, Mycolicibacterium frederiksbergense IN53 (formerly Mycobacterium frederiksbergense IN53), Dietzia sp. IN133, Pseudomonas sp. IN132, Arthrobacter sp. IN212, and Gordonia sp. IN138) came from the hydrocarbon-degrading microbial collection of the Department of Microbiology (at the Oil and Gas Institute–National Research Institute, Poland). The strains were isolated from temperate (all except IN212) and alpine (IN212) hydrocarbon-exposed soils. In addition, IN53 was previously tested alone or as a member of a hydrocarbon-degrading consortium [32]. The hydrocarbon-metabolizing capabilities of these bacteria were examined by growth on mineral medium supplemented with the tested compounds (see Table 6), and according to the methods of Wrenn and Venosa [82]. Strain diagnostic features were determined based on microscopic observations, morphology, growth on the selective agar media, and biochemical profile (API tests, bioMerieux). The strains were phylogenetically identified by 16S rDNA sequencing analysis as described previously [17]. The 16S rDNA sequences of these strains have been deposited in the NCBI database and their accession numbers are shown in Table 6.

Pure cultures of the individual strains were grown in a BD Difco™ nutrient broth (Difco, USA) supplemented with sodium acetate (0.2%, *w*/*v*) and incubated at room temperature with shaking at 150 rpm for 24–72 h to obtain density 10^8^–10^9^ cfu/mL. A mixed culture (designated C2), used in this study, was constructed by mixing equal volumes of each strain. 

### 4.4. Experimental Design

The soil samples used in this study were collected from a historically heavily hydrocarbon-polluted G58 waste-pit area located in an oil and gas plant in Grabownica (southeast Poland). The contamination of this site resulted from storing drilling wastes on the site for over 50 years. Surface samples from the top 20 cm of the soil profile were taken from several non-overlapping areas (20 cm width × 20 cm length). The characteristics of the soil were as follows: pH of 6.1, initial moisture content 15.3%, NH_4_^+^ nitrogen: 302.1 mg/g d.w. soil, NO_3_^−^ nitrogen: 95.1 mg/g d.w. soil, PO_4_^3−^ phosphate: 76.2 mg/g d.w. soil, and phenols: 21.8 mg/g d.w. soil; TAH and PAH contents were 17 757.2 ± 1175.2 and 2777.5 ± 211.2 mg/g d.w. soil, respectively. The total number of heterotrophic aerobic bacteria was 8.7 ± 4.3 × 10^5^ cfu/g d.w. soil.

The experimental setup was designed to assess the hydrocarbon removal (TAHs and PAHs) under different bioremediation treatments (Table 7). Each of the laboratory microcosms were prepared by filling a sterile glass jar with 500 g of contaminated soil. The soil moisture content was adjusted and maintained at 25%, and the incubation temperature was kept at 20–25 °C. Once–twice a week, each microcosm was aerated, and the moisture level was maintained using deionized water. The jars were plugged with sterile cotton stoppers to maintain oxygen access. There were two controls: (1) HgCl_2_-treated soil as a reference to assess abiotic hydrocarbon loss, and (2) a microcosm without any amendment to estimate the biodegradation potential of the native soil community (Table 7).

Biostimulation consisted of (1) the addition of inorganic NP fertilizer as nutrient amendments to obtain a molar ratio 7:1 of N and P, and (2) correction of the pH value via calcium carbonate addition (final pH 7.5). Bioaugmented microcosms (BA-C1 and BA-C2) were also biostimulated as above and then inoculated (both inoculants had been diluted shortly before inoculation took place) with 0.5 mL/g d.w. soil of the C1 or C2, respectively. The final quantity of introduced C1 and C2 was (2.4 ± 1.8) × 10^6^ and (3.3 ± 2.4) × 10^6^ cfu/mL, respectively. Thus, after introduction, the final number of microbial cells of C1 and C2 was presumably within the range 10^5^–10^6^ cfu/g d.w. soil. Each treatment was performed in quadruplicate. After 60 days of incubation, the residual hydrocarbon content was assessed using GC/FID (gas chromatography with a flame ionization detector) analyses performed according to the previously described methods [13,17]. Furthermore, after that period, toxicity, mutagenicity, and genetic (metagenomic and metatranscriptomic) analyses were conducted. The toxic capacities of the analyzed soils were evaluated using commercial sets, namely: The Phytotoxkit^TM^ test (MicroBioTests Inc., Nazareth, Belgium), the Ostracodtoxkit(F)^TM^ (MicroBioTest Inc., Nazareth, Belgium), and the Microtox^®^ Solid Phase Test (SDI, Newark, DE, USA). The mutagenic potential of the residual soil contamination was assessed by the commercial version of the Ames test (Muta-ChromoPlate^TM^ Kit (EBPI, Mississauga, ON, Canada). The details regarding the tests’ performances are included in Supplemental Experimental Procedure.

### 4.5. DNA Extraction, PCR and Illumina MiSeq-Based Sequencing

Total DNA was extracted using Genomic Mini AX Bacteria kit (A&A Biotechnology, Gdynia, Poland) according to the manufacturer’s protocol. For soil samples, additional mechanical lysis with zirconium beads was performed using FastPrep-24 instrument (MP Biomedicals). After isolation, the DNA from each replicate was pooled and then purified using an Anty-Inhibitor Kit (A&A Biotechnology, Poland). The DNA quality and concentration were verified by electrophoresis and fluorometry (Qubit 4 Fluorometer, Thermofisher Scientific, USA). Library preparation and sequencing was performed by Genomed S.A. (Warsaw, Poland). The V3-V4 hypervariable region of the gene encoding for bacterial 16S rRNA was amplified using a 341F and 785R primer set [83]. Each library was prepared in a two-step PCR protocol based on the Illumina 16 metagenomic library prep guide, using Q5 Hotstart High-Fidelity DNA Polymerase (NEBNext High-Fidelity 2xPCR Master Mix; New England, BioLabs) and a Nextera Index kit (2 × 250 bp). Paired-end sequencing was performed on a MiSeq platform (Illumina) with a MiSeq Reagent kit v2 following the manufacturer’s run protocol. Automatic analysis and de-multiplexing of the raw reads were performed on MiSeq with the use of a MiSeq Reporter (MSR) ver. 2.6. Amplicon sequence variants (ASVs) were extracted using the DADA2 ver. 1.12 package [84] in R ver. 3.6.1 (R Core Team, 2016). The reads were quality-filtered and trimmed using the function filterAndTrim with the following parameters: truncLen = c (245,245), maxN = 0, maxEE = (2,5), truncQ = 2, and trimLeft = (17,21). The error rates were estimated by *learnErrors*, where the *nbases* parameter was set to 2·× 10^8^. Sequences were dereplicated using *derepFastq* with default parameters, and exact sequence variants were resolved using *dada*. Chimeric sequences were removed using the DADA2 function “removeBimeraDenovo” with method = ”consensus” and “MinFoldParentOverAbundance = 16”. In this step, sequences were identified as chimeric and removed. The taxonomy was assigned to ASVs using the Naïve Bayesian classifier on the DADA2-formatted reference SILVA 16S rRNA database ver. 132 [85] with the minimum bootstrap confidence of 50. Multiple sequence alignment of the ASVs was performed using DECIPHER R package ver. 2.12. [86], and a phylogenetic tree was constructed using phangorn R package ver. 2.5.5 [87]. The frequency table, taxonomy assignment and phylogenetic tree information were used to create a phyloseq object, and bacterial community analyses were performed using phyloseq R package ver. 1.28.0 [88]. The alpha diversity metrics were calculated using the “estimate_richness” function in the phyloseq.

### 4.6. Metatranscriptome Sequencing

The total RNA was extracted using the Total RNA Mini Kit (A&A Biotechnology, Gliwice, Poland), according to the manufacturer’s protocol. rRNa removal was performed by using the Illumina Ribo-Zero rRNA Removal kit (Bacteria). cDNA libraries were generated using rRNA-depleted RNA with the NEBNext^®^ Ultra RNA Library Prep Kit (NEB, Ipswich, MA, USA), following the manufacturer’s instructions. The libraries were sequenced on the Illumina HiSeq4000 platform using a paired-end (2 × 100 bp) sequencing strategy at the Genomed S.A. (Warsaw, Poland). All of the paired-end reads were combined to decrease sequencing errors and submitted directly to MG-RAST (Metagenome Rapid Annotation using Subsystem Technology pipeline ver. 4.0.3, [89] for automated annotation and further analyses. Quality control was performed on sequences in MG-RAST, including dereplication, ambiguous base filtering, quality filtering, and length filtering. All data were trimmed by removing uninformative and/or duplicative reads before any further studies. Details regarding quality filtering are included in Supplementary Materials: Table S1. Annotation of the metatranscriptome sequences was performed using the standard parameters for sequence quality control. The data were compared to the SEED Subsystem using a maximum e-value of 10^−5^, a minimum identity of 60%, and a minimum alignment length of 15 measured in aa for protein and bp for RNA databases. Additionally, the alkane monooxygenase related sequences identified based on MG-RAST BLAT were extracted and applied to blastx search query against the NCBI-nr database at an e-value cut-off of 10^−5^. The blastx results were analyzed with a MEGAN ver. 6 [90] to assign a taxonomic affiliation of the transcripts using a threshold of bitscore > 50.

### 4.7. Statistical Analyses

The statistical data analyses were conducted with the free PAST software ver. 3.26 [91]. The obtained data were first tested for normal distribution. Then, they were analyzed either with a one-way ANOVA followed by a post-hoc pairwise Tukey test (when the ANOVA produced significant results) or with a Kruskal–Wallis H test followed by a Mann–Whitney U test with Bonferroni correction (when the Kruskal–Wallis test produced significant differences). This approach was also employed for statistical analysis of the metagenomic data since only composite samples were analyzed.

### 4.8. Data Availability

The raw prokaryotic and metatranscriptomics sequences reported here were deposited in the NCBI GenBank database under BioProject accession number PRJNA577076. Raw metatranscriptomic sequences were uploaded to the MG-RAST server and the annotated data are available under MG-RAST ID number mgm4861089.

## 5. Conclusions

An environmentally friendly bioaugmentation method was shown to significantly increase removal of hydrocarbons (TAHs and PAHs) from contaminated soil microcosms. The best results were achieved by the application of the defined mixed culture consisting of well-characterized, hydrocarbon-degrading bacterial strains, while both biostimulation itself and bioaugmentation with undefined community were much less efficient. All three treatments impacted the composition of the indigenous prokaryotic microbial community. Removal of highly complex hydrocarbon contamination, such as in the analyzed soil, requires various catabolically versatile bacteria which are functionally active and temporarily persistent in a given habitat. Sixty days after the introduction of allochthonous organisms as mixed culture C2, some members did not survive. Others were still active but their abundance was also relatively low. Therefore, the persistence of introduced bacteria limited to the duration of the bioremediation process should be beneficial for the native community. Our study also demonstrated that ecotoxicity and mutagenicity bioassays are useful, complementary tools for chemical analyses to evaluate bioremediation efficiency. To conclude, the application of a defined mixed culture, where each member reveals broadened hydrocarbon-degrading capabilities, could be an interesting alternative for bioremediation purposes of soil contaminated with aliphatic compounds and high PAH load. The certainty that all strains possess required catabolic features is an evident advantage over an undefined microbial community. Consequently, large volumes of ready-to-use mixed culture can be easily obtained by growing bacteria on nutrient-rich media. This approach should be especially beneficial for large-scale bioremediation projects where time is a limiting factor.

## Figures and Tables

**Table 1:** The abundance pattern of dominant amplicon sequence variants (ASVs) in the community C1. Only ASVs detected at a frequency > 1% are shown.

ASV-ID	Class	Genus	Relative Abundance
ASV15	Gammaproteobacteria	*Alcaligenes*	27%
ASV1	Alphaproteobacteria	*Pseudochrobactrum*	22%
ASV35	Alphaproteobacteria	*Aquamicrobium*	12%
ASV66	Firmicutes	*Enterococcus*	12%
ASV47	Alphaproteobacteria	*Brevundimonas*	9%
ASV366	Firmicutes	*Alkaliphilus*	4%
ASV475	Firmicutes	*Clostridium sensu stricto 16*	2%
ASV516	Firmicutes	*Clostridioides*	2%
ASV673	Firmicutes	*Melissococcus*	1%
ASV622	Actinobacteria	*Leucobacter*	1%

**Table 2:** Initial and residual contents of total aliphatic hydrocarbons (TAHs), unidentified hydrocarbons, alkanes (∑*n*C_8_–nC_22_ and ∑*n*C_23_–*n*C_35_) and polycyclic aromatic hydrocarbons (∑PAHs), unidentified PAHs, and distinguished PAH groups. The data are presented as the mean values ± SD (*n* = 4).

Hydrocarbons	Content ± SD (mg/kg d.w. soil)				
	Initial (0 Day)	After 60 Days			
		Control	BS	BA-C1	BA-C2
Total aliphatic hydrocarbons (TAHs)	17757.2 ± 1175.2 ^AB^	17467.2 ± 1049.5 ^CD^	11372.0 ± 699.8 ^E^	5290.9 ± 297.7 ^ACF^	2310.3 ± 126.2 ^BDEF^
Unidentified aliphatic hydrocarbons	5496.6 ± 304.5	5512.6 ± 286.0	3779.2 ± 245.4	1647.5 ± 108.2	707.3 ± 49.9
∑*n*C_8_–*n*C_22_	9324.1 ± 655.3 ^GH^	8983.8 ± 564.1 ^IJ^	5274.2 ± 316.9 ^K^	2254.5 ± 109.6 ^GIL^	861.2 ± 39.3 ^HJKL^
∑*n*C_23_–*n*C_35_	2936.5 ± 215.4 ^M^	2917.0 ± 199.4 ^N^	2283.7 ± 137.5 ^O^	1388.9 ± 79.9	741.8 ± 37.0 ^MNO^
∑PAHs	2777.5 ± 211.2 ^a^	2785.6 ± 162.4 ^b^	2120.8 ± 118.6 ^c^	988.7 ± 54.3	411.6 ± 21.9 ^abc^
Unidentified PAHs	455.8 ± 32.7	483.2 ± 24.1	387.2 ± 19.8	229.4 ± 11.9	99.7 ± 4.5
∑Two- and three-ring PAHs	1459.5 ± 207.6 ^de^	1413.2 ± 182.9 ^fg^	1000.0 ± 129.6 ^h^	329.6 ± 41.3 ^df^	112.0 ± 14.7 ^egh^
∑Four- and five- ring PAHs	829.1 ± 79.0 ^i^	856.4 ± 86.5 ^j^	701.95 ± 62.0 ^k^	401.7 ± 26.6	177.4 ± 6.6 ^ijk^
∑Six-ring PAHs	32.2 ± 3.9	32.8 ± 3.3	31.7 ± 3.3	28.1 ± 2.5	22.5 ± 1.4

Control: untreated microcosms, BS: biostimulated microcosms, BA-C1 and BA-C2: microcosms bioaugmented with the bacterial community C1 and the mixed culture C2, respectively. Letters (A–O and a–k) indicate the statistically significant differences between treatments.

**Table 3:** Values of biodegradation indices in the different microcosms. The data are presented as the mean values ± SD (*n* = 4).

Microcosms	*n*C_17_/Pristane	*n*C_18_/Phytane
Control	9.0 ± 0.6	8.2 ± 0.8
BS	5.2 ± 0.4	4.5 ± 0.5
BA-C1	2.0 ± 0.2	1.6 ± 0.2
BA-C2	0.7 ± 0.05	0.5 ± 0.05

Control: untreated microcosms, BS: biostimulated microcosms, BA-C1 and BA-C2: microcosms bioaugmented with the bacterial community C1 and the mixed culture C2, respectively.

**Table 4:** Bioassay results.

Microcosms	Ostracodtoxkit Test		Microtox Solid Phase Test	Ames Test
	Mortality (%) (Chronic Toxicity)	Growth Inhibition (%) (Chronic Toxicity)	Toxicity (TU)	Mutagenicity Ratio
Control	54.6 ± 5,1	No data	28.7 ± 2.9	14.2
BS	40.3 ± 3.2	44.5 ± 4.3	22.9 ± 2.2	10.5
BA-C1	33.1 ± 3.2	36.5 ± 3.5	13.2 ± 1.3	7.2
BA-C2	17.0 ± 0.9	20.4 ± 1.3	1.2 ± 0.2	1.2

Control: untreated microcosms, BS: biostimulated microcosms, BA-C1 and BA-C2: microcosms bioaugmented with the bacterial community C1 and the mixed culture C2, respectively.

**Table 5:** Richness and diversity of bacterial communities in the analyzed soil samples.

Indices	Control	BS	BA-C1	BA-C2
Observed richness	1676	1335	1190	1279
Shannon index	5.08	4.82	4.73	5.55
Simpson	0.98	0.97	0.96	0.99
Chao-1	2111.52	1608.19	1379.15	1481.41
ACE	2163.65	1605.98	1356.98	1439.74

Control: untreated microcosms, BS: biostimulated microcosms, BA-C1 and BA-C2: microcosms bioaugmented with the bacterial community C1 and the mixed culture C2, respectively.

**Table 6:** Hydrocarbon-degrading capabilities of bacterial strains comprising the mixed culture C2.

Strain	NCBI Accession Number	*n*C_18_H_38_	iso-C_19_H_40_	TOL, XYL	NAP	ANT	PHEN	FLU	FLUO	PYR
*Rhodococcus erythropolis* IN119	KT923331	+	+	+	+	−	−	−	−	−
*Mycolicibacterium frederiksbergense* IN53	JN572675	+	+	−	+	+	+	−	−	+
*Dietzia* sp. IN133	KT923300	+	+	+	+	−	−	−	−	−
*Pseudomonas* sp. IN132	KT923299	+	+	+	+	+/−	+	+/−	+/−	+/−
*Arthrobacter* sp. IN212	KT923314	+	+	+	+/−	+/−	+/−	−	−	−
*Rhodococcus* sp. IN136	KT923330	+	+	−	+	−	−	−	+	−
*Gordonia* sp. IN138	KT923297	+	+	+	+	+/−	−	−	−	−

iso-C_19_H_40_: pristane, TOL: toluene, XYL: mixture of xylenes, NAP: naphthalene, ANT: anthracene, PHEN: phenanthrene, FLU: fluorene, FLUO: fluoranthene, PYR: pyrene, +: growth, −: no growth, +/−: ambiguous observation.

**Table 7:** Summary of the experimental design.

Treatment	Treatment Details	Purpose
abiotic control	HgCl_2_-treated soil (sterilized soil)	abiotic control
control	soil	control (natural attenuation)
BS	soil + inorganic N, P	biostimulation
BA-C1	soil + inorganic N, P + consortium C1	biostimulation and bioaugmentation
BA-C2	soil + inorganic N, P + mixed culture C2	biostimulation and bioaugmentation

## Supplementary Materials

The following are available online, Supplemental Experimental Procedures, Figure S1: The results of MEGAN analysis demonstrating taxonomic assignment of BLAST hits of the reads associated with alkane monooxygenase, to the NCBI nr database. Table S1: Information regarding sequence quality control of the analyzed metatranscriptomic data.
