# Supplementary material for: Hydrocarbon Removal by Two Differently Developed Microbial Inoculants and Comparing Their Actions with Biostimulation Treatment
Source: Molecules. 2020 Feb 4;25(3):661. doi: 10.3390/molecules25030661 (PMC7036810; doi:10.3390/molecules25030661)
Supplement: Supplementary file 1 [file molecules-25-00661-s001.zip › Supplementary material.pdf]

Supplementary material

Hydrocarbon Removal by Two Differently Developed Microbial Inoculants and Comparing Their Actions with Biostimulation Treatment

Joanna Brzeszcz [1,*,†], Piotr Kapusta [1,*,†], Teresa Steliga [2] and Anna Turkiewicz [1]

[1] Department of Microbiology, Oil and Gas Institute–National Research Institute, 31-503 Krakow, ul. Lubicz 25A, Poland; joanna.brzeszcz@inig.pl (J.B.); piotr.kapusta@inig.pl (P.K.); anna.turkiewicz@inig.pl (A.T.)

[2] Department of Reservoir Fluid Production Technology, Oil and Gas Institute–National Research Institute, 31-503 Krakow, ul. Lubicz 25 A, Poland; teresa.steliga@inig.pl

* Correspondence: joanna.brzeszcz@inig.pl(J.B.); piotr.kapusta@inig.pl (P.K.); Tel.: +48-12-617-76-89 (J.B.); +48-12-617-76-90 (P.K.)

† These authors contributed equally to this work.

Supplemental Experimental Procedures

Phytotoxkit™ test

In this bioassay, both the decrease/absence of seed germination and the decrease of early root growth after 3-day exposure of the seeds to contaminated soils were measured. The seeds of three different plants were used, namely, seeds of monocotyl sorgho (*Sorghum saccharatum*) and dicotyls garden cress (*Lepidium sativum*) and mustard (*Sinapis alba*). Tests were performed according to the producer's instructions. In brief, soil was placed on polystyrene testing plates and soaked with water until the total water capacity was reached. The plates were covered with filter paper and seeds of the mentioned plants. Incubation was performed at 25 °C in the dark for 72 hours. After incubation, the number of germinated seeds and the lengths of roots were measured. Then, the percent of seed germination inhibition and the percent of root growth inhibition were calculated according to the formula: $I = \frac{(A-B)}{A} \cdot 100\%$, where I is inhibition (%), A is the seed germination or root length in the reference control soil supplied by the Phytotoxkit™ producer, and B is the seed germination or root length in the analyzed soils (i.e. control, BS, BA-C1, BA-C2).

Ostracodtoxkit(F)™

A chronic "direct contact" bioassay with benthic ostracod *Heterocypris incongruens* was performed according to the standard procedure of the Ostracodtoxkit(F)™. The analyzed soil was placed in each well of the 6-well plastic microplate, previously filled with 2 mL of standard freshwater. After adding 2 mL of algal food suspension, ten 52-hour hatched neonates were transferred gently into each well. Incubation was performed in the dark at 25 °C for 6 days. Two toxic effect endpoints were reported: ostracod mortality and ostracod growth inhibition. Growth inhibition was calculated according to the following formula: $GI = 100\% - (\frac{A}{B} \cdot 100\%)$, where A and B refer to the ostracod length in the analyzed and reference soils, respectively.

Microtox® Solid Phase Test

The Microtox® Solid Phase Test (SDI, USA) is an acute toxicity test using the luminescent bacterium, *Vibrio fischeri*. A reduction in its luminescent ability during exposure to contaminants is taken as a measure of toxicity. Briefly, 2.0 g of soil was added to 100 mL of deionised water; then, the solution was mixed until turning a dark color. Bacteria were then incubated with the sample for 15 min. After that, microorganisms were separated from the soil suspensions and the bioluminescence was measured using the DeltaTox Analyzer. The results were reported as EC_{50}, which represents the effective concentration of the soil sample that will reduce 50% of bacterial light emissions at a given time. To simplify, the EC_{50} values were converted to toxicity units (TU) according to the following formula: $TU = \frac{100}{EC_{50}} \cdot 100\%$.

Ames test

This bioassay was performed using histidine-dependent bacterial strains of the *Salmonella typhimurium* strain TA100 to detect base-pair substitutions. The samples were tested without metabolic activation (S9) to detect direct mutagenicity. The mentioned strains are characterized by gene mutation that leads to the incapability of L-histidine synthesis. Exposure of this microorganism to mutagens can result in the reversal of mutation. Revertant bacteria are detected by their ability to grow on a medium deprived of the compound required by the parent test strains. The presence of mutagenic compounds may cause an increase in the number of revertant colonies relative to the background levels. The bioassay was performed according to the manufacture's protocol. In brief, the lyophilized bacteria were cultured overnight in a nutrient broth (delivered by the biotest producer) at 37 °C. The contaminants were extracted using dichloromethane, evaporated, dissolved in dimethyl sulfoxide (DMSO), and filter sterilized. Then, the contaminants were diluted, mixed with bacterial inoculum, and incubated at 37 °C for 48 h prior to counting the number of revertant colonies. Sodium azide was used as a positive control and water as a negative one.

Table S1. Information regarding sequence quality control of the analyzed metatranscriptomic data.

MG-RAST accession number	mgm4861089
Count of uploaded sequences	304 412 346
Sequence count after MG-RAST quality control	16 725 623
Average sequence length (bp)	100 ± 1bp
Mean GC content (%)	58±13 %
