# Supplementary figures and images for: Hydrocarbon Removal by Two Differently Developed Microbial Inoculants and Comparing Their Actions with Biostimulation Treatment
Source: Molecules. 2020 Feb 4;25(3):661. doi: 10.3390/molecules25030661 (PMC7036810; doi:10.3390/molecules25030661)

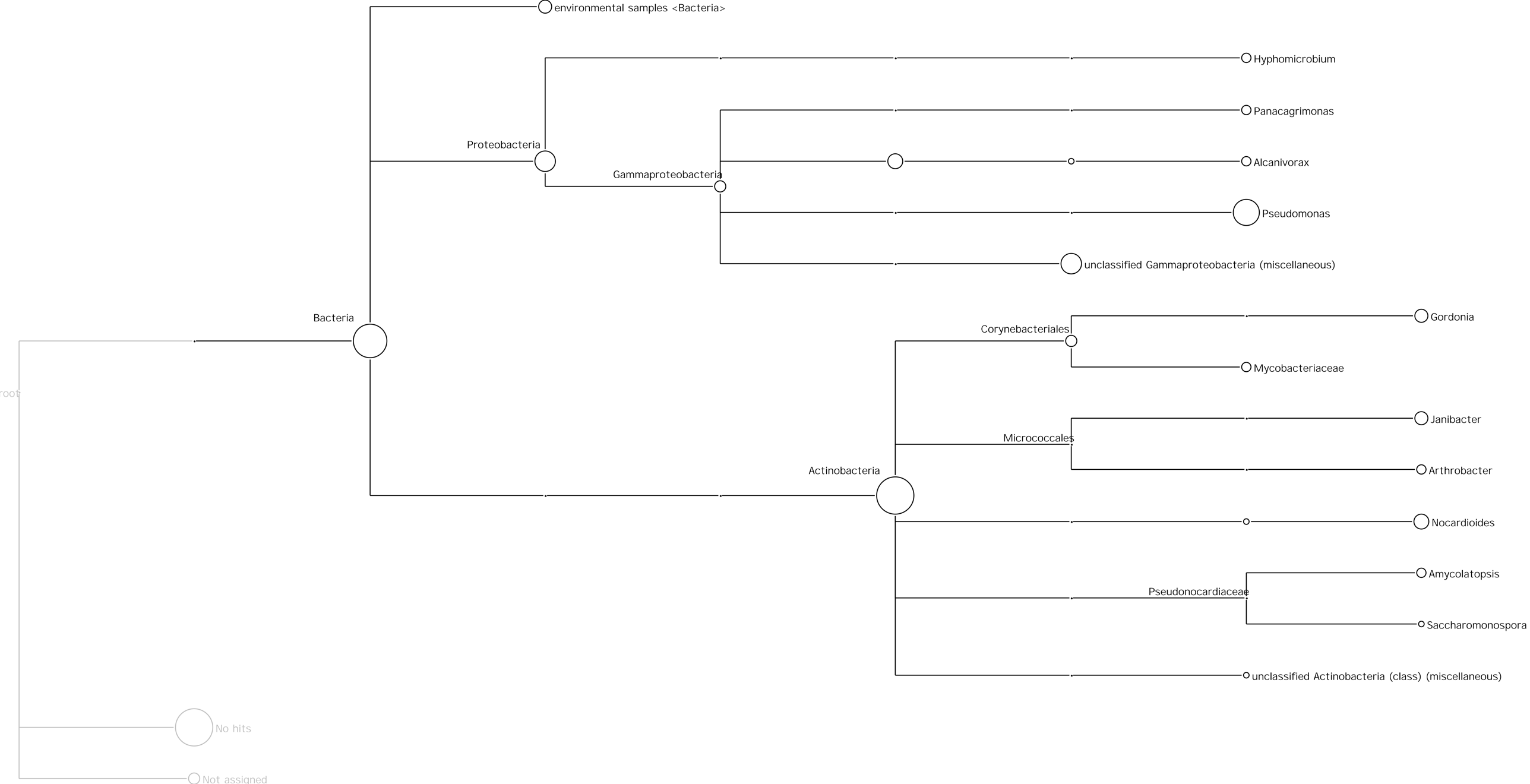

Supplement: Supplementary file 1 [file molecules-25-00661-s001.zip › Figure S1.pdf]
